# Genome-Wide Identification, Molecular Evolution, and Expression Divergence of Aluminum-Activated Malate Transporters in Apples

**DOI:** 10.3390/ijms19092807

**Published:** 2018-09-18

**Authors:** Baiquan Ma, Yangyang Yuan, Meng Gao, Tonghui Qi, Mingjun Li, Fengwang Ma

**Affiliations:** State Key Laboratory of Crop Stress Biology for Arid Areas/Shaanxi Key Laboratory of Apple, College of Horticulture, Northwest A and F University, Yangling 712100, China; bqma87@nwsuaf.edu.cn (B.M.); yy.yuan@nwsuaf.edu.cn (Y.Y.); gaomeng086630@gmail.com (M.G.); QTH913@nwafu.edu.cn (T.Q.)

**Keywords:** apple, *ALMT*, gene duplication, expression profile, subcellular localization

## Abstract

Aluminum-activated malate transporters (*ALMTs*) play an important role in aluminum tolerance, stomatal opening, and fruit acidity in plants. However, the evolutionary pattern of the *ALMT* gene family in apples remains relatively unknown. In this study, a total of 25 *MdALMT* genes were identified from the apple reference genome of the “Golden Delicious” doubled-haploid tree (GDDH13). The physiological and biochemical properties, gene structure, and conserved motifs of *MdALMT* genes were examined. Chromosome location and gene-duplication analysis indicated that whole-genome duplication/segmental duplication played an important role in the expansion of the *MdALMT* gene family. The Ka/Ks ratio of duplicated *MdALMT* genes showed that members of this family have undergone strong purifying selection. Through exploration of the phylogenetic relationships, seven subgroups were classified, and higher old gene duplication frequency and significantly different evolutionary rates of the *ALMT* gene families were detected. In addition, the functional divergence of *ALMT* genes occurred during the evolutionary process of *Rosaceae* species. Furthermore, the functional divergence of *MdALMT* genes was confirmed by expression discrepancy and different subcellular localizations. This study provides the foundation to better understand the molecular evolution of *MdALMT* genes and further facilitate functional analysis to unravel their exact role in apples.

## 1. Introduction

Genes encoding aluminum-activated malate transporters (*ALMT*s) are widespread, are commonly found in plants, and play a vital role in the transport of organic acids across membranes. Organic acids play a crucial role in plants’ primary metabolism and are involved in adaptation to local environments, such as stomatal movement, aluminum tolerance, pH regulation, and response to stress [1,2]. During the past two decades, various studies have indicated that the exudation of organic acids (especially malic acid) can regulate plant tolerance to metal, mitigate nutritional stress, and define fruit acidity [1,2,3,4]. Thus, deciphering the exact mechanism(s) of organic acid transport in plants should improve our understanding of plant tolerance to biotic and abiotic stresses.

The toxicity of aluminum ions (Al^3+^) is a significant limiting factor for agricultural crop production on acidic soils. Several plants release di- and tricarboxylic acids that act as chelating agents, immobilizing phytotoxic Al^3+^. The binding of Al^3+^ to di-/tricarboxylic acids leads to the formation of stable, nontoxic complexes, preventing Al^3+^ from entering the cells of the roots [5,6,7]. Anion channels need to be involved to support the efflux of organic acids, such as citrate or malate effluxes that transport acids from the cytosol to the apoplast against the electrochemical gradient. The Al^3+^-dependent plasma-membrane anion was first identified and characterized in wheat-root protoplasts from the Al^3+^-tolerant line by Ryan et al. [3]; further studies indicated that extracellular Al^3+^ could activate these types of channels. To date, the functions of *ALMT* gene members are highly diverse, and their roles transcend beyond the function in root Al^3+^-resistance responses to a variety of other physiological processes. *AtALMT*1 was proven to be one of 14 *ALMT* genes in *Arabidopsis thaliana* involved in Al^3+^ tolerance [8], and its expression can be clearly induced by indole-3-acetic acid, abscisic acid, low pH, and hydrogen peroxide treatments [9]. *AtALMT6* and *AtALMT9* are localized on the tonoplast of guard cells and are involved in the increase in malic acid content in vacuoles [10,11]. *AtALMT9* is a chloride channel. It is activated by physiological concentrations of cytosolic malate and plays an important role in stomatal opening [12]. In contrast, *AtALMT6* is regulated by changes in cytosolic Ca^2+^, and *Atalmt6* plants exhibit reduced malate concentrations in vacuoles compared with wild-type *Arabidopsis*; however, the mutation does not have obvious phenotypic differences [11]. In addition, heterologous overexpression in yeast of *Ma1*, a member of the *ALMT* gene family in apples, can increase the malic acid content in the vacuole [4]. Similarly, electrophysiological analyses indicated that *VvALMT9* mediates the selective flux of malate and tartarate into the vacuole in grape berries [13]. Thus, functional divergence of *ALMT* genes during the species evolution of plants resulted in altered functional constraints, and it is critical to evaluate the evolutionary relationship between different species.

Gene duplication and selection are two important factors that influence plant morphogenetic evolution. After gene duplication, one copy of the gene may become nonfunctional (gene death), acquire a novel function (neofunctionalization), or take on a separate function (subfunctionalization) [14]. In general, most duplicated genes accumulate deleterious mutations and become nonfunctional, and a subset of duplicated genes evolves novel functions that are eventually retained under positive selection [14,15]. The evolutionary pattern of duplicated genes needs to be clarified during the plant-speciation process. In recent years, the evolutionary pattern of duplicated genes has been investigated. For example, Chen et al. [16] revealed that strong positive gene selection drives rapid diversification of R-genes in *Arabidopsis* species relatives, and Li et al. [17] reported unique evolutionary patterns in a number of gramineous NBS-LRR genes. In addition, Wang et al. [18] found that divergent evolutionary patterns of transporter genes are involved in sugar accumulation.

The domesticated apple (*Malus* × *domestica* Borkh.) is one of the most important fruit crops in temperate regions of the world. Recently, the genome of the domesticated apple was fully sequenced based on the doubled haploid GDDH13 [19]. This information provides an opportunity to further analyze the *ALMT* gene family in apple. In this study, we identified 25 *ALMT* genes in the apple genome and investigated their phylogenetic relationships, gene duplication, evolutionary rates, selective patterns, and functional divergence. Furthermore, the expression pattern and subcellular localization of *MdALMT* genes were also investigated. Our results reveal the molecular characteristics and evolutionary pattern of the *MdALMT* gene family and provide a foundation for future elucidation of the biological functions of *ALMT* genes in apples.

## 2. Results

### 2.1. Identification and Characterization of ALMT Gene Family in Apples

In this study, a total of 25 putative *MdALMT* genes were identified in the apple genome. The physiological and biochemical properties of the *MdALMT* proteins were characterized, and the general information for the 25 *MdALMT* genes is shown in Table 1. The length of the MdALMT proteins ranged from 284 to 812 amino acids, with predicted molecular weights of 31.61 kDa to 90.91 kDa. The theoretical isoelectric point (pI) of the MdALMT proteins ranged from 5.97 to 9.55, and the grand average of hydropathicity (GRAVY) ranged from −0.174 to 0.357. In addition, the instability index of the MdALMT proteins ranged from 25.08 to 43.50, and the aliphatic index ranged from 86.51 to 107.91. Furthermore, the conserved transmembrane domain (TMD) was investigated, and 6 to 7 TMDs were detected in the N-terminus of the ALMT proteins.

**Figure 1:**
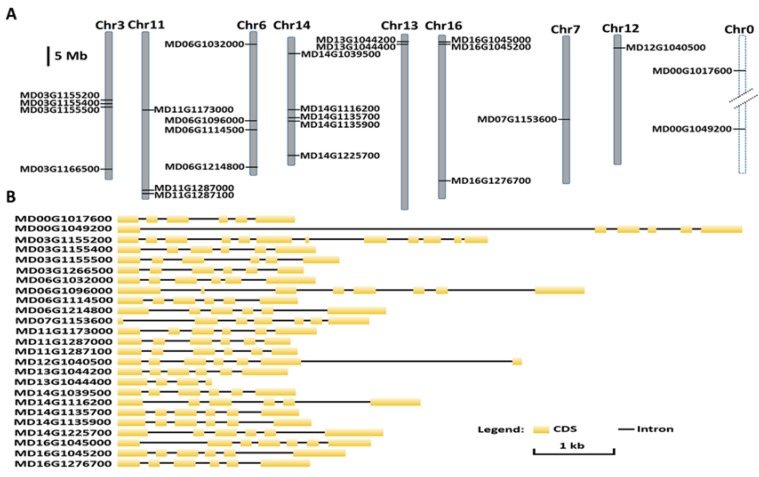
Genes encoding aluminum-activated malate transporter (*ALMT*) in the apple genome. (**A**) Chromosomal location of apple *ALMT* genes. The dashed line indicates the unanchored sequences; (**B**) genome organization of apple *ALMT* genes. Yellow indicates exons; black line indicates introns.

Genomic structural analysis showed that the exon number of each *MdALMT* gene ranged dramatically, from 4 to 12 (Figure 1B). The majority of *MdALMT* genes consisted of six exons, while three *MdALMT* genes, MD07G1153600, MD12G1040500, and MD16G1045000, contained seven exons, whereas MD03G1155200 contained 12 exons, MD06G1096000 contained 8 exons and MD13G1044400 contained 4 exons. This suggests that exon loss and gain have both occurred in the *ALMT* gene family. To further systematically discover the motif of *MdALMTs*, we estimated the distribution of conserved motifs using the online MEME server. The results showed that 10 putative conserved motifs were identified in all of the MdALMT proteins except MD13G1044400, and the distribution of conserved motifs is presented in Figure S1. Thus, the *MdALMTs* clearly exhibited extreme conservation during the evolutionary process.

### 2.2. Chromosome Distribution and Duplication of MdALMT Genes

The genome distribution of the 25 *MdALMT* genes is shown in Figure 1A. Of these genes, 21 were located on three homologous pairs of chromosomes (3–11, 6–14, and 13–16), one was located on chromosome 7, one was located on chromosome 12, and two were located on chromosome 0 (unanchored sequences). The largest number of *MdALMT* genes (five) was detected on chromosome 14, whereas the fewest were found on chromosome 7 and 12 (one per chromosome). Four genes were detected on both chromosomes 3 and 6, while three genes were found on both chromosomes 11 and 16.

**Figure 2:**
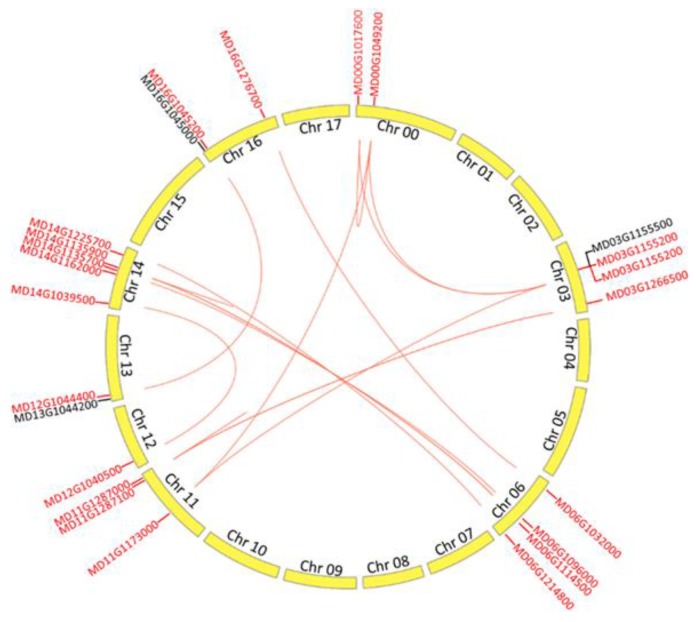
Chromosomal distribution and gene duplication of the *MdALMT* genes. Gene IDs are labeled on the basis of their position on the chromosomes. The red line represents the putative whole-genome-duplication (WGD)/segmental-duplication genes or tandem-duplication genes.

It has been confirmed that whole-genome duplication and segmental duplication occurred during the process of apple domestication [20]. Subsequently, whole-genome duplications and segmental duplications (WGD/segmental duplication) of the *MdALMT* genes were analyzed. As shown in Figure 2, a total of 22 (88%) *ALMT* genes in apples exhibited WGD/segmental duplication. These observations suggest that WGD/segmental duplication played an important role in the expansion of the apple *ALMT* gene family because this process allows the retention of numerous duplicated genes in the genome. In addition, three pairs of genes, MD00G1017600-MD00G1049200, MD14G1135700-MD14G1135900, and MD11G1287000-MD11G1287100, were regarded as tandem-duplicated genes. The *Ka* (nonsynonymous), *Ks* (synonymous), and the *K*a/*K*s ratio (*ω*) were used to estimate evolutionary rates and selective pressure [21]. In general, *ω* > 1 indicates positive selection, *ω* < 1 provides evidence of purifying selection, and *ω* = 1 supports the hypothesis of neutral evolution. To better understand the selective pressures acting on duplicated *MdALMT* genes, the ω value of duplicated gene pairs was calculated (Table S2). The ω value of all *MdALMT* gene pairs was less than 1, indicating that their evolution occurred under the influence of strong purifying selection. Thus, we concluded that the evolutionary pattern of *MdALMT* genes was conserved during apple domestication.

### 2.3. Phylogenetic Analysis, Gene Duplication, and Gene Loss in Four Rosaceae Species

**Figure 3:**
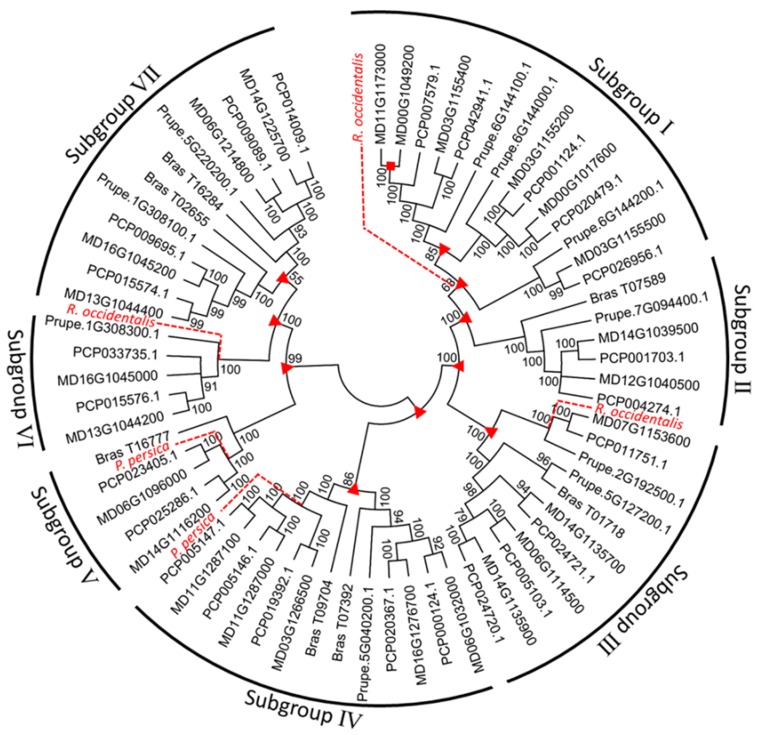
Phylogenetic tree analysis of genes encoding *ALMT* in Rosaceae using the neighbor-joining (NJ) method. Recent and old duplication events are indicated by the red square and triangle, respectively. Bootstrap values (higher than 50%) are shown near branched lines. Gene loss is indicated by red dash line.

A phylogenetic tree of *ALMT*s was constructed to analyze the evolutionary relationship among ALMT proteins from apples (*M. domestica*), peaches (*Prunus persica*), pears (*Pyrus communis*), and black raspberries (*Rubus occidentalis*), using neighbor-joining (NJ) and maximum-likelihood (ML) methods that yielded very similar topologies. Based on the phylogenetic relationships, the ALMT proteins could be classified into seven subgroups (Figure 3). All subgroups clustered with bootstrap values above 50%. Separation of genes from each *Rosaceae* species was clearly observed in each subgroup. Of the 25 *MdALMT* genes, eight, two, four, five, two, two, and four genes were assigned to subgroups I, II, III, IV, V, VI, and VII, respectively.

Depending on the evolutionary timeline, gene duplication can be categorized as old or recent duplication [22]. We categorized duplication as recent if it took place in the species (species–species duplication), whereas duplication was categorized as old if the event occurred prior to the radiation of *Rosaceae* species [18]. We tested for signs of old and recent duplication in each *ALMT* gene subgroup in our analysis (Figure 3) and identified ten incidences of old duplication and one incidence of recent duplication, indicating that all *ALMT* genes, except MD11G1173000-MD00G1049200, existed prior to the *Rosaceae* species split. In addition, it is interesting to note that old duplication was detected in subgroups I, III, IV, and VII. This suggests that these subgroups underwent at least one duplication event before the *Rosaceae* species split.

Recent gene loss was observed in five subgroups (subgroups I, III, IV, V, and VII), in *R. occidentalis* and *P. persica* (Figure 3). However, we did not detect signs of old gene loss of the *ALMT* genes.

### 2.4. Positive Selection Analysis of ALMT Genes in Four Rosaceae Species

**Figure 4:**
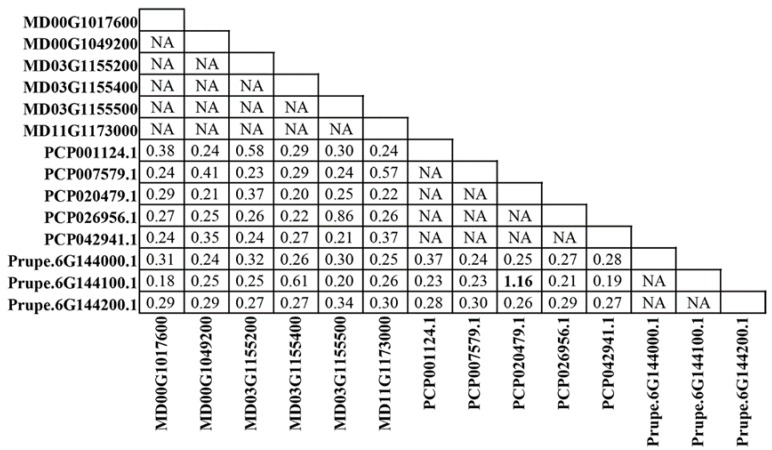
Estimation of *ω* values of *MdALMT* gene subgroup I using the maximum-likelihood (ML) method. The *ω* value higher than 1 is highlighted in black bold.

**Figure 5:**
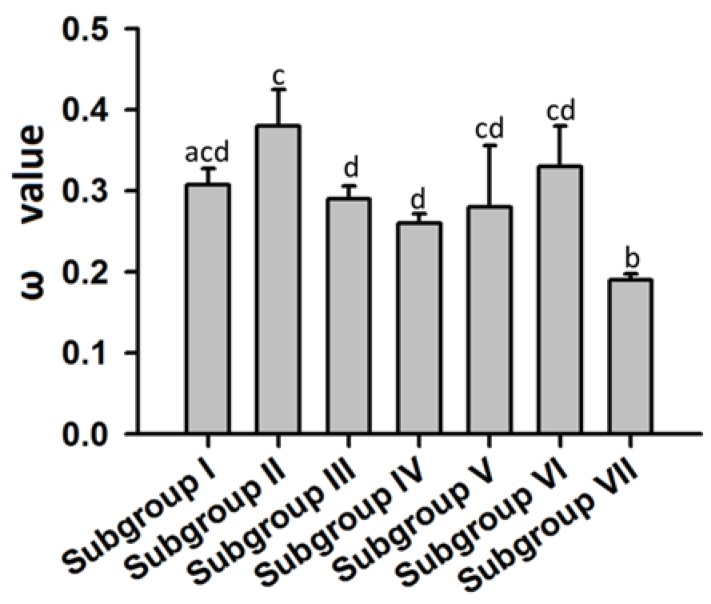
Mean ω values of *ALMT* genes in different subgroups. Different lowercase letters indicate significant differences among subfamilies (*t*-test, LSD test at *p* < 0.01). Error bars correspond to SE of means.

To detect whether the *ALMT* genes in each subgroup were subject to different evolutionary constraints, we calculated pairwise ω values for all tested *ALMT* genes using a pairwise comparison model (Figure 4, Table S3). Next, we compared the pairwise ω values of the *ALMT* genes in each subgroup. The average ω values of the *ALMT* genes in each subgroup displayed the following pattern among the seven subgroups: subgroup II > subgroup VI > subgroup I > subgroup III > subgroup V > subgroup IV> subgroup VII (Figure 5). In contrast, *ALMT* genes from subgroup VII showed significantly lower ω values than the other groups (*p* < 0.01), whereas the ω values of *ALMT* genes in subgroup II were significantly higher than those in subgroups III and IV (Figure 5). Overall, pairwise ω values indicated a significant difference in the evolutionary rates of the *ALMT* genes in each subgroup. In addition, we compared the *ω* values between members of subgroup I. Interestingly, positive selection was detected in one pair of orthologous genes (PCP020479.1 and Prupe.6G144100.1), with a ratio of 1.16.

To characterize the evolution rate since the most recent common ancestor of the *ALMT* subgroups, a branch-specific model was used to detect variations in evolutionary constraints throughout different *ALMT* subgroups. As shown in Table 2, *ALMT* subgroups V and VI + VII had a *p*-value lower than 0.05, while others had a *p*-value higher than 0.05. This indicated that the evolutionary rate differed significantly between *ALMT* subgroups V and VI + VII.

### 2.5. Estimation of Functional Divergence

The pairwise coefficient of type-II functional divergence (θ_II_) between subgroups was calculated using DIVERGE v3.0; a θ_II_ value larger than zero indicated a degree of functional divergence between two subgroups. As shown in Table 3, the θ_II_ values were pairwise compared among *ALMT* subgroups. All pairwise comparisons showed that θ_II_ values were significantly less than 0 in subgroups I/II and II/III, suggesting that these genes did not diverge significantly with respect to function. However, the θ_II_ values of other pairwise comparisons were higher than 0, suggesting that type II functional divergence of *ALMT* genes occurred during the evolutionary process of Rosaceae species. Our findings suggest that *ALMT* genes exhibited both functional conservation and divergence during *Rosaceae* species evolution.

### 2.6. Different Expression Patterns and Subcellular Positions of ALMT Genes

**Figure 6:**
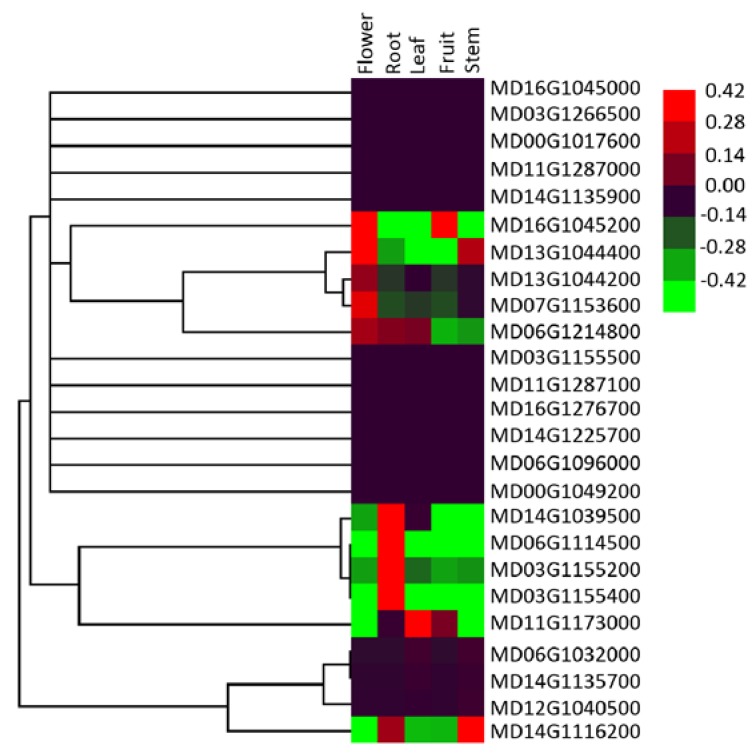
Expression profiles of apple *ALMTs* in five kinds of tissue, full-bloom flower (Flower), Root, mature leaf (Leaf), ripening fruit (Fruit), and Stem. Heat map was constructed based on relative expression levels. Different colors represent different expression level, with red representing the highest value of gene expression.

**Figure 7:**
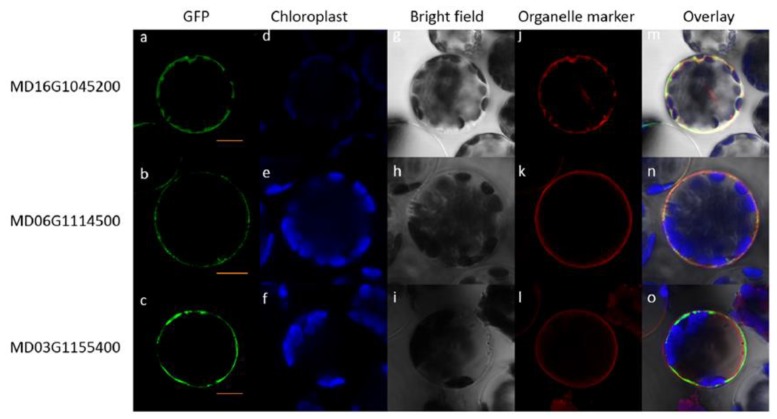
Subcellular localization of MD16G1045200, MD06G1114500, and MD03G1155400 proteins in *Arabidopsis* mesophyll protoplasts. First column (**a**–**c**) shows the fluorescence of the MD16G1045200-GFP, MD06G1114500-GFP, and MD03G1155400-GFP fusion protein, respectively. Second column (**d**–**f**) shows chloroplast autofluorescence. Third column (**g**–**i**) shows a bright-field image of each protoplast. **j**: Fluorescence of vac-rk (vacuolar marker) protein. **k**,**l**: Fluorescence of pm-rk (plasma-membrane marker) protein. Fifth column (**m**–**o**) represents an overlay of the fluorescent images. Bars = 5 μm.

To further investigate the expression patterns of the *ALMT* gene family in apples, we evaluated the expression profiles of *ALMT* genes in five kinds of apple tissue: full-bloom flower, root, mature leaf, ripening fruit, and stem (Figure 6, Table S4). The expression pattern of apple *ALMT* genes showed significant divergence among different kinds of tissue. Of the 25 apple *ALMT* genes, 14 were expressed at low levels in all five kinds of tissue, with relative expression levels ranging from 0 to 0.1. In contrast, one apple *ALMT* gene (MD11G1173000) showed high expression levels in all tested tissue. Additionally, MD16G1045200, which belongs to subgroup VII, was highly expressed in flower and fruit; however, its homologs, MD03G1155400 and MD06G1114500, in subgroup I and III, respectively, showed high expression levels in roots. Subcellular localization of MD16G1045200, MD06G1114500, and MD03G1155400 was determined in *Arabidopsis* mesophyll protoplasts (Figure 7). Confocal microscopy indicated that the MD16G1045200-GFP protein resided in the tonoplast, whereas the MD06G1114500-GFP and MD03G1155400-GFP proteins were targeted to the cell membrane. These findings indicate that the expression discrepancy and different subcellular localizations of apple *ALMT* genes are consistent with the fact that functional divergence occurred in subgroups I/VII and III/VII, not in subgroup I/III (Table 3).

## 3. Discussion

It is well known that *ALMT* genes play a vital role in a variety of physiological processes, such as mineral nutrition, metal-toxicity tolerance, fruit acidity, and guard-cell movement. In many species, the members of the *ALMT* family have been identified and characterized. For example, in the model plant *A. thaliana*, 14 *ALMT* genes have been identified [2]. The recent release of a high-quality de novo assembly of the apple genome, which used the latest sequencing and optical-mapping technologies, provided an opportunity to identify the *MdALMT* gene family. In this study, a total of 25 *MdALMT* genes were identified in the apple genome through a BLAST search (Figure 1); results are inconsistent with the findings of previous reports [4,23]. This discrepancy might be because the new apple reference genome of the “Golden Delicious” doubled-haploid tree (GDDH13) was used in our study, while the genome sequence of the apple cultivar “Golden Delicious” was used in the study reported by Ma et al. [4] and Xu et al. [23].

The apple is a diploidized autopolyploid species with a basic chromosome number of *x* = 17 [20,24]. Of the 25 *MdALMT* genes, 21 are located on three homologous pairs of chromosomes. For example, chromosomes 13 and 16 are homologous-pair sequences and both contain a cluster of two *MdALMT* genes on the top chromosome. Similar findings were also obtained for homologously paired chromosomes 3 and 11, and 6 and 14. Furthermore, we investigated the duplication of *MdALMT* genes, and 22 *MdALMT* genes exhibited WGD/segmental duplication in the apple genome (Figure 2). These results indicated that the duplication of *MdALMT* genes is related to WGD/segmental duplication during the process of apple speciation and domestication. In addition, chromosomes 3 and 11 are homologous pairs of chromosomes. Chromosome 3 contains a single *MdALMT* gene (MD03G1166500) on the bottom chromosome, while only two *MdALMT* genes (MD11G1287000 and MD11G1287100) were detected on the homologous region of chromosome 11. Similarly, chromosomes 6 and 14 are homologous pairs. A cluster of two *MdALMT* genes, MD14G1135700 and MD14G1135900, was found on middle chromosome 14, while only a single *MdALMT* gene MD06G1114500 is located on the homologous region of chromosome 6. These results indicate that tandem duplication of *MdALMT* genes likely occurred on chromosomes 11 and 14 (Figure 2). It is worth noting that a single *MdALMT* gene was detected on chromosome 7, but no *MdALMT* gene was found on chromosomes 1 and 2, which are homologous to chromosome 7 [20]. Similar results were observed for chromosome 12 (Figure 1). Duplicated gene copies after WGD/segmental duplication showed rapid divergence and gene loss [25]. Thus, we speculate that *MdALMT* genes on chromosomes 7 and 12 may have been lost in the apple ancestor because chromosomes 3 and 11 are known homologous pairs [20,24], and two *MdALMT* genes (MD00G1017600 and MD00G1049200) exhibited WGD/segmental duplication (Figure 2). We presume that the *MdALMT* genes on chromosome 0 (MD00G1017600 and MD00G1049200) are likely to be located on chromosome 11.

In this study, a phylogenetic analysis revealed that 25 *MdALMT* genes with other *ALMT* genes from three *Rosaceae* species were classified into seven subgroups (Figure 3), and the *MdALMT* genes grouped with *ALMT* genes from the pear (*P. communis*), indicating a close relationship between *ALMT* genes from apples and pears. Gene duplication is considered to be the primary basis of the evolution of novel functions of proteins. In the course of evolution, some polyploidy-derived duplicated genes have been retained, whereas others have lost their function [26]. To better understand the evolution of the *ALMT* gene family in apples, the genomic structure and conserved motifs of *MdALMT* genes were characterized. Most of the *MdALMT* genes exhibited similar numbers of exons and conserved motifs (Figure 1B, Figure S1). Furthermore, the ω value is an important measure of genetic differentiation [18], and the ω value of all the duplicated *MdALMT* gene pairs was less than 1 (Table S2), indicating a slow evolutionary rate and purifying selection during apple evolution and domestication.

Gene duplication followed by gene death, subfunctionalization, and/or neofunctionalization deeply impacts the evolution of proteins and these processes are regarded as the major sources of adaptive functional novelty in eukaryotes [14,15]. In our study, functional divergence of the *ALMT* gene family was calculated using Divergence v3.0 software. The values of the pairwise coefficient of type-II functional divergence were significantly less than 0 in subgroups I/II and II/III, and higher than 0 in other pairwise comparisons (Table 3). Furthermore, the expression profile of *MdALMT* genes was determined, which showed rapid divergence in expression (Figure 6). Three *MdALMT* genes (MD16G1045200, MD03G1155400, and MD06G1114500), which belong to different subgroups, have acquired divergent expression patterns. MD16G1045200 was highly expressed in flower and fruit, but its homologs, MD03G1155400 and MD06G1114500, showed high expression levels in roots. The subcellular localization of these three *MdALMT* genes was also determined (Figure 7). The MD16G1045200-GFP protein resides in the tonoplast, whereas the MD06G1114500-GFP and MD03G1155400-GFP proteins are targeted to the cell membrane. *ALMT* genes are an important family of anion channels, involved in the efflux of organic acids in plants. Thus, we speculate that the functional divergence of *ALMT* genes depends on their tissue-specific expression and subcellular localization.

## 4. Materials and Methods

### 4.1. Identification of ALMT Genes and Phylogenetic Analyses

The amino acid sequence of *ALMT*s in *A. thaliana* [10] was retrieved from TAIR (https://www.arabidopsis.org/), and used as a query sequence to identify homologous genes by basic local-alignment search-tool searches of the reference genome sequence of the apple [19], peach [27], pear [28], and black raspberry [29] according to the procedure described by Tatusov et al. [30] and Wang et al. [18]. Briefly, BLAST 2.2.24 software (http://blast.ncbi.nlm.nih.gov/Blast.cgi) was used to construct a local BLAST database for each species and the all-against-all protein BLAST, where putative orthologous gene pairs were identified by reciprocal best similarity matching; this analysis was carried out to identify homologous genes. ClustalX2 was used to perform multiple alignments of the amino acid sequences; the resulting dataset was used to construct a phylogenetic tree with the help of MEGA v.7 software (https://www.megasoftware.net/) with both the NJ and ML methods [31]. In the NJ method, the following parameters were used: bootstrap, 1000 replicates; *p*-distance; and pairwise deletion. In the ML method, the parameters were: bootstrap, 1000 replicates; Jones–Taylor–Thornton; partial deletion; and branch-swap filter, very strong.

### 4.2. Gene Structure and Conserved Motif Analysis of ALMTs

The exon–intron organization of *MdALMT* genes was determined by aligning coding sequences with the corresponding genomic sequences. Diagrams were generated using Gene Structure Display Server 2.0 (http://gsds.cbi.pku.edu.cn/) and adjusted manually, as deemed necessary. Conserved motifs of *MdALMT* genes were identified and analyzed using the online MEME suite server (http://meme-suite.org/). The parameters were set as follows: maximum numbers of different motifs, 10; minimum width, 10; and maximum width, 50.

### 4.3. Syntenic Analysis of ALMTs in Apple

The duplicated *ALMT* genes in the apple were identified using MCScanX as previously described by Wang et al. [32]. Briefly, all the protein sequences from apples were compared using BLASTP (http://www.ncbi.nlm.nih.gov/blast/blast.cgi) with an *e*-value less than 1 × 10^−5^. The BLASTP outputs with gene-location files were used as an input for MCScanX to identify syntenic gene pairs and duplication types with default settings. Circos-069-3 software (http://www.circos.ca/software/download/circos/) was used to construct the schematic diagram of the putative duplication of *MdALMT* genes, and the putative WGD/segmental-duplicated genes or tandem-duplicated genes were connected by links [33].

### 4.4. Gene Duplication and Gene-Loss Estimations

Using the phylogenetic analysis approach, gene duplication and gene loss were estimated by manually checking each subgroup. It was assumed that each gene subgroup comprised at least one member from each species. If there were two or more subgroups, or more than one member of each species, it was considered to have one or more duplications. If a member was not found in one or more species of each subgroup, it was considered gene loss. Each identified gene loss was further confirmed by searching the GenBank database using BLASTP.

### 4.5. Detection of Positive Selection

Amino acid sequences were aligned using Clustal X2. Multiple alignments of proteins and the corresponding coding sequences were submitted to PAL2NAL (http://www.bork.embl.de/pal2nal/). The output result was used to estimate the *ω* values between the different branches of the phylogenetic tree using a branch-specific algorithm in Codeml program from PAML v.4.9 (http://abacus.gene.ucl.ac.uk/software/paml.html) [34]. In brief, the alternative-likelihood models were used to investigate the evolutionary rates between different branches, 1-ratio model with a single *ω* value (dN/dS ratio) estimated for all branches, while 2 independent *ω* values (dN/dS ratio) were assumed among different branches (the background and the foreground branches) in the 2-ratio model. The significant difference between 2 models was estimated based on twice the log-likelihood difference (2∆ln*L* = 2(ln*L*_1_ − ln*L*_0_), and the optimized ω values were determined using the likelihood-ratio test (LRT).

The coding sequence of *ALMT*s was aligned using Clustal X2 and was further adjusted manually. The resulting data were used to calculate the ω value with the help of the KaKs Calculator package v2.0 (https://sourceforge.net/projects/kakscalculator2/) using the maximum-likelihood method [35]. A pairwise comparison was used to test the variation of the ω values of *ALMT*s among the different branches of the phylogenetic trees. Briefly, the ω values between two randomly selected sequences within each subgroup were calculated with the help of the KaKs Calculator. Statistical analysis was carried out using SPSS 17.0 (SPSS Inc., Chicago, IL, USA), and a *t*-test was used to estimate significance of differences in ω values of *ALMT*s between different subgroups.

### 4.6. Detection of Functional Divergence after Gene Duplication

CLUSTAL X2 was used to align the amino acid sequences of *ALMT*s, and the results were stored in FASTA format. PHYLIP v3.6 software (http://evolution.gs.washington.edu/phylip.html) was used to assemble the NJ phylogenetic tree. DIVERGE v3.0 software (Iowa State University, Ames, USA) was used to examine type-II functional divergence of the *ALMT* subgroup after gene duplication [36]. Each gene subgroup within the same phylogenetic tree was considered to be one cluster.

### 4.7. RNA Isolation and Quantitative RT-PCR (qRT-PCR) Analysis

Five kinds of apple tissue, i.e., full-bloom flower, root, mature leaf, ripening fruit, and stem, were selected for quantitative RT-PCR assay. Total RNA was extracted using the Wolact Plant RNA Isolation Kit (Wolact, Hongkong, China) according to the manufacturer’s instructions, and first-strand cDNA was synthesized using TransScript One-Step gDNA Removal and cDNA Synthesis SuperMix (TRANS, Beijing, China) following the manufacturer’s instructions. qRT-PCR with a SYBR Green I Master Mix (TaKaRa, Dalian, China) was performed on an Applied Biosystems 7500 Real-Time PCR System (Applied Biosystems, Foster City, CA, USA). Relative expression level of each gene was measured according to the cycle threshold (Ct), also known as the 2^−ΔΔCT^ method, and all the analyses consisted of 3 biological replicates. An actin gene described in a previous study [4], was selected as a constitutive control, and all the primers used for qRT-PCR are listed in Table S1.

### 4.8. Subcellular Localization of Apple ALMTs in Arabidopsis Protoplasts

To localize apple ALMTs (MD16G1045200, MD06G1114500, and MD03G1155400) at the subcellular level, constitutively expressed MD16G1045200-GFP, MD06G1114500-GFP, and MD03G1155400-GFP fusion proteins were created. Three pairs of primers (5-ATGGCGGCCAAAATCGGG-3/5-TTAGTTCTTCAACCGCAAACTCCTAAA-3, 5-ATGGAGTGGAGGATAAAAATGGCC-3/5-TCAGACCCTTCGGAGGGCAT-3, and 5-ATGGAGATCACCCAGTTAGAAACTGC-3/5-TTACGCTTCCTCCCTTGGTTTTG-3) were used to amplify the entire coding regions of MD16G1045200, MD06G1114500, and MD03G1155400, respectively. PCR (polymerase chain reaction) products was purified, and inserted into cloning vector pEASY-Blunt. Then, the full coding sequences of MD16G1045200, MD06G1114500, and MD03G1155400 were inserted into the pHBT-GFP-NOS vector under the control of the cauliflower mosaic virus 35S promoter. The organelle markers, which contained the mCherry reporter gene, for plasma membrane (pm-rk) and vacuole (vac-rk) were used as a control [37], and were performed in cotransformation experiments with MD16G1045200-GFP, MD06G1114500-GFP, and MD03G1155400-GFP constructs. Subcellular localization of fusion proteins was assessed with PEG-calcium mediated transfection in *Arabidopsis* protoplasts [38]. The fluorescent signal was detected in *Arabidopsis* protoplasts after 12–24 h past transfection using confocal laser-scanning microscope Leica TCS SP8 (Leica, Germany).

## 5. Conclusions

This work integrated phylogenetic, gene-structure, gene-duplication, molecular-evolution, expression, and subcellular-localization analyses to provide a deep understanding of the evolutionary pattern of *ALMT* genes in apples. In this study, a total of 25 *MdALMT* genes were identified in the apple reference genome of the “Golden Delicious” doubled-haploid tree (GDDH13). All *MdALMT* genes contain similar genomic structures and conserved motifs. Chromosome location and gene duplication of *MdALMT* gene analysis indicated that WGD/segmental duplication and tandem duplication occurred during apple domestication, and WGD/segmental duplication was involved in the expansion of the apple *ALMT* gene family. The Ka/Ks ratio of duplicated *MdALMT* genes showed that *MdALMT* genes have undergone strong purifying selection.

The *ALMT* genes from four *Rosaceae* species, apple, peach, pear, and black raspberry, were used to construct the phylogenetic trees, and seven subgroups were classified. Based on phylogenetic relationships, higher old duplication frequency and significant differences in the evolutionary rates of *ALMT* genes were observed. In addition, the functional divergence of *ALMT* genes occurred during the evolutionary process of *Rosaceae* species. Furthermore, expression profiling of *MdALMT* genes was performed, which showed significant divergence among five types of apple tissue. According to the subcellular localization, *MdALMT* genes were targeted to the cell membrane or tonoplast. Our results indicated that the functional divergence of apple *ALMT* genes was confirmed by expression discrepancy and different subcellular localizations.

## Figures and Tables

**Table 1:** Summary information of physiological and biochemical properties of the MdALMT proteins.

Gene ID	Animo Acids	MW (kDa)	pI	GRAVY	Instability Index	Aliphatic Index	TMD
MD13G1044200	521	58.60	8.30	0.066	26.07	91.71	6
MD07G1153600	498	54.52	7.93	0.135	28.12	101.43	6
MD13G1044400	284	31.61	8.86	0.357	25.08	104.01	6
MD14G1116200	546	61.23	6.69	−0.125	40.61	91.43	6
MD06G1096000	719	80.62	7.18	−0.106	33.56	86.51	7
MD14G1135700	497	54.36	7.09	0.158	27.06	96.20	7
MD06G1114500	488	53.70	7.88	0.148	31.09	95.16	7
MD03G1155400	484	53.33	8.83	0.048	41.05	94.50	6
MD11G1173000	485	53.83	9.39	0.041	36.04	95.32	6
MD03G1155200	812	90.91	9.55	−0.174	43.50	92.60	6
MD14G1039500	494	53.86	8.75	0.221	34.37	107.19	6
MD12G1040500	532	57.95	8.76	0.164	32.81	104.10	6
MD03G1155500	467	51.96	8.39	0.046	35.46	96.30	6
MD16G1045200	568	63.85	5.97	0.012	36.38	93.19	7
MD16G1045000	600	66.51	6.64	0.131	28.26	94.12	7
MD06G1214800	603	67.64	7.92	−0.095	43.33	89.40	6
MD14G1225700	597	66.89	7.55	−0.080	38.72	89.82	6
MD06G1032000	541	60.31	8.17	0.057	34.71	98.61	6
MD16G1276700	522	58.68	7.99	−0.007	34.82	98.30	6
MD14G1135900	496	54.49	8.22	0.183	30.68	99.33	7
MD03G1266500	423	46.88	8.09	0.145	39.44	106.71	6
MD11G1287000	433	48.22	8.31	0.164	39.89	106.03	6
MD11G1287100	430	47.61	6.56	0.224	36.35	107.91	6
MD00G1017600	472	51.81	8.21	0.151	43.26	97.52	6
MD00G1049200	485	53.69	9.28	0.066	36.40	97.94	6

MW: Molecular weight of the amino acid sequence; pI: Theoretical isoelectric point; GRAVY: Grand average of hydropathicity; TMD: Transmembrane domains.

**Table 2:** Likelihood-ratio test (LRT) statistic and parameters from Branch model of PAML.

Subgroup	Null Hypothesis		Alternative Hypothesis			LRT	
	-In L	*ω*	-In L	*ω*1	*ω*2	Statistic	*p*
I + II	8024.95	0.27	8024.38	0.25	0.29	1.14	>0.05
(I + II) + III	7860.32	0.28	7860.08	0.28	0.30	0.48	>0.05
(I + II + III) + VI	9497.94	0.25	9496.27	0.26	0.21	3.34	>0.05
VI + VII	3210.45	0.22	3210.00	0.21	0.25	0.90	>0.05
(VI + VII) + V	4121.21	0.20	4114.04	0.22	0.08	14.34	<0.01

**Table 3:** Estimation of type II functional divergence (θ) using type II of DIVERGE software.

Subgroup A	Subgroup B	θ-II Value
I	II	0.22 ± 0.05
	III	−0.28 ± 0.16
	IV	0.21 ± 0.13
	V	0.62 ± 0.04
	VI	0.51 ± 0.05
	VII	0.87 ± 0.30
II	III	−0.25 ± 0.11
	IV	0.43 ± 0.04
	V	0.53 ± 0.07
	VI	0.65 ± 0.03
	VII	0.52 ± 0.06
III	IV	0.13 ± 0.10
	V	0.46 ± 0.07
	VI	0.25 ± 0.12
	VII	0.24 ± 0.11
IV	V	0.57 ± 0.03
	VI	0.55 ± 0.04
	VII	0.21 ± 0.11
V	VI	0.52 ± 0.05
	VII	0.45 ± 0.05
VI	VII	0.26 ±0.06

## Supplementary Materials

Supplementary materials can be found at http://www.mdpi.com/1422-0067/19/9/2807/s1.
