# Supplementary figures and images for: Genome-Wide Identification, Molecular Evolution, and Expression Divergence of Aluminum-Activated Malate Transporters in Apples
Source: Int J Mol Sci. 2018 Sep 18;19(9):2807. doi: 10.3390/ijms19092807 (PMC6163302; doi:10.3390/ijms19092807)

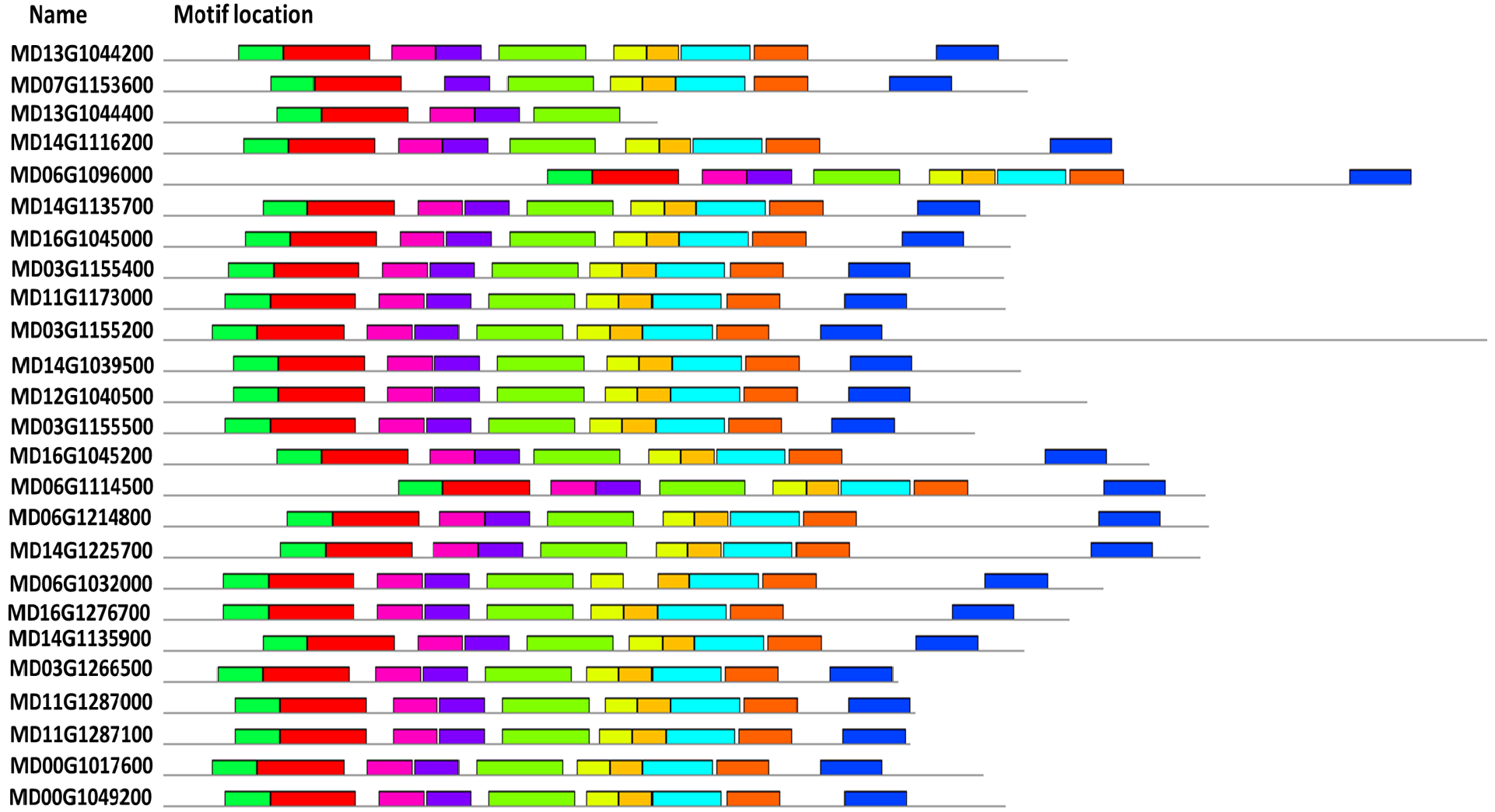

Supplement: Supplementary file 1 [file ijms-19-02807-s001.zip › Supplimentary/Fig.S1.tif]
